# Ethyl 1-*sec*-butyl-2-(4-chloro­phen­yl)-1*H*-benzimidazole-5-carboxyl­ate

**DOI:** 10.1107/S1600536810033945

**Published:** 2010-08-28

**Authors:** Natarajan Arumugam, Aisyah Saad Abdul Rahim, Hasnah Osman, Ching Kheng Quah, Hoong-Kun Fun

**Affiliations:** a School of Pharmaceutical Sciences, Universiti Sains Malaysia, 11800 USM, Penang, Malaysia; b School of Chemical Sciences, Universiti Sains Malaysia, 11800 USM, Penang, Malaysia; c X-ray Crystallography Unit, School of Physics, Universiti Sains Malaysia, 11800 USM, Penang, Malaysia

## Abstract

In the title compound, C_20_H_21_ClN_2_O_2_, the ethyl 1*H*-benzimidazole-5-carboxyl­ate ring system, excluding the methyl­ene and methyl H atoms, is almost planar, with a maximum deviation of 0.055 (1) Å, and makes a dihedral angle of 40.63 (4)° with the benzene ring. The *sec*-butyl group is disordered over two positions, with refined site occupancies of 0.855 (4) and 0.145 (4). In the crystal, mol­ecules are linked into chains along [010] *via* inter­molecular C—H⋯O hydrogen bonds and are further inter­connected by C—H⋯Cl inter­actions into two-dimensional networks parallel to (001). The crystal structure is further consolidated by C—H⋯π inter­actions.

## Related literature

For the synthesis of the title compound, see: Arumugam *et al.* (2010*a* ▶,*b* ▶,*c* ▶). For general background to and the therapeutic properties of benzimidazole derivatives, see: Bonfanti *et al.* (2008 ▶); Evans *et al.* (1997 ▶); Hori *et al.* (2002 ▶); Snow (2007 ▶). For a related structure, see: Eltayeb *et al.* (2009 ▶). For reference bond lengths, see: Allen *et al.* (1987 ▶). For the stability of the temperature controller used in the data collection, see: Cosier & Glazer (1986 ▶).
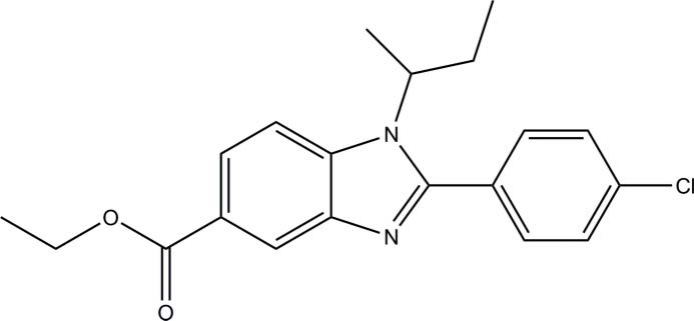


## Experimental

### 

#### Crystal data

C_20_H_21_ClN_2_O_2_
*M* *_r_* = 356.84
Monoclinic, 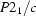

*a* = 10.4321 (9) Å
*b* = 12.6218 (12) Å
*c* = 13.6896 (12) Å
β = 97.043 (3)°
*V* = 1788.9 (3) Å^3^
*Z* = 4
Mo *K*α radiation
μ = 0.23 mm^−1^
*T* = 100 K
0.45 × 0.34 × 0.10 mm

#### Data collection

Bruker SMART APEXII DUO CCD area-detector diffractometer
Absorption correction: multi-scan (*SADABS*; Bruker, 2009 ▶) *T*_min_ = 0.904, *T*_max_ = 0.977
21234 measured reflections
8437 independent reflections
6651 reflections with *I* > 2σ(*I*)
*R*_int_ = 0.038

#### Refinement

*R*[*F* ^2^ > 2σ(*F* ^2^)] = 0.050
*wR*(*F* ^2^) = 0.175
*S* = 1.09
8437 reflections
248 parameters
H-atom parameters constrained
Δρ_max_ = 0.75 e Å^−3^
Δρ_min_ = −0.39 e Å^−3^

### 

Data collection: *APEX2* (Bruker, 2009 ▶); cell refinement: *SAINT* (Bruker, 2009 ▶); data reduction: *SAINT*; program(s) used to solve structure: *SHELXTL* (Sheldrick, 2008 ▶); program(s) used to refine structure: *SHELXTL*; molecular graphics: *SHELXTL*; software used to prepare material for publication: *SHELXTL* and *PLATON* (Spek, 2009 ▶).

## Supplementary Material

Crystal structure: contains datablocks global, I. DOI: 10.1107/S1600536810033945/wn2406sup1.cif

Structure factors: contains datablocks I. DOI: 10.1107/S1600536810033945/wn2406Isup2.hkl

Additional supplementary materials:  crystallographic information; 3D view; checkCIF report

## Figures and Tables

**Table 1:** Hydrogen-bond geometry (Å, °) *Cg*1 is the centroid of the N1/C7/N2/C13/C8 ring.

*D*—H⋯*A*	*D*—H	H⋯*A*	*D*⋯*A*	*D*—H⋯*A*
C12—H12*A*⋯O2^i^	0.93	2.57	3.4994 (16)	174
C16—H16*A*⋯Cl1^ii^	0.96	2.73	3.4651 (17)	134
C19*A*—H19*B*⋯*Cg*1	0.96	2.71	3.3457 (18)	124

Symmetry codes: (i) 
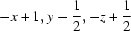
; (ii) 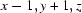.

**Fig. 1.**
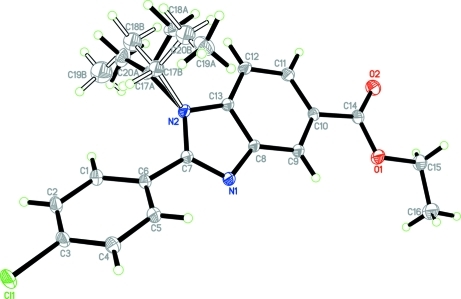
The molecular structure of the title compound showing 50% probability displacement ellipsoids for non-H atoms and the atom-numbering scheme. Both disorder components are shown; atom labels with suffix A correspond to the major disorder component, while those with suffix B correspond to the minor disorder component.

**Fig. 2.**
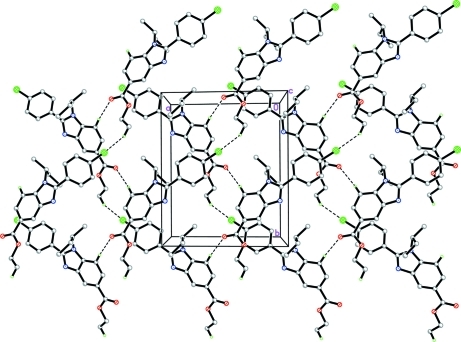
The crystal structure of the title compound, viewed along the c axis. H atoms not involved in intermolecular interactions (dashed lines) and the minor disorder component have been omitted for clarity.

## supplementary crystallographic information

### Comment

Benzimidazole is an important pharmacophore in drug discovery and its derivatives have shown various therapeutic properties such as antiviral (Bonfanti *et al.*, 2008) and anti-HIV-1 (Evans *et al.*, 1997) activities. Benzimidazoles are used as biomimetics of guanine residues and selectively inhibit endothelial cell growth and suppress angiogenesis *in vitro* and *in vivo* (Hori *et al.*, 2002). In particular, substituted benzimidazole derivatives are also inhibitors of tyrosine kinase and are useful for inhibiting cell proliferation for the treatment of cancer (Snow *et al.*, 2007). In view of their importance, the crystal structure determination of the title compound was carried out and the results are presented here.

The molecular structure of the title compound is shown in Fig. 1. The ethyl 1*H*-benzimidazole-5-carboxylate ring system (O1/O2/N1/N2/C7–C16), excluding methylene and methyl H atoms, is almost planar, with a maximum deviation of 0.055 (1) Å for atom O2, and makes a dihedral angle of 40.63 (4)° with the benzene (C1–C6) ring. The *sec*-butyl group is disordered over two positions with refined site-occupancies of 0.855 (4) and 0.145 (4). The bond lengths (Allen *et al.*, 1987) and angles in the title compound are within normal ranges and comparable to those in a related crystal structure (Eltayeb *et al.*, 2009).

In the crystal structure (Fig. 2), the molecules are linked into one-dimensional chains along [010] *via* intermolecular C12—H12A···O2 hydrogen bonds and are further interconnected by C16–H16A···Cl1 interactions into two-dimensional networks parallel to (001). The crystal structure is further consolidated by C—H···*Cg*1 (Table 1) interactions; *Cg*1 is the centroid of the N1/C7/N2/C13/C8 ring.

### Experimental

The title compound was synthesized using previously reported procedures (Arumugam *et al.*, 2010**a*,*b*,c*). The crude product was recrystallized from ethyl acetate to yield the title compound as colourless crystals.

### Refinement

All H atoms were positioned geometrically and refined using a riding model, with C—H = 0.93–0.98 Å and *U*_iso_(H) = x*U*_eq_(C), where x = 1.5 for methyl H and 1.2 for all other H atoms. A rotating-group model was applied for the methyl groups. The *sec*-butyl group attached to the benzimidazole ring system is disordered over two positions, with refined site-occupancies of 0.855 (4) and 0.145 (4).

### Crystal data

**Table:**

C_20_H_21_ClN_2_O_2_	*F*(000) = 752
*M**_r_* = 356.84	*D*_x_ = 1.325 Mg m^−^^3^
Monoclinic, *P*2_1_/*c*	Mo *K*α radiation, λ = 0.71073 Å
Hall symbol: -P 2ybc	Cell parameters from 5929 reflections
*a* = 10.4321 (9) Å	θ = 2.8–36.1°
*b* = 12.6218 (12) Å	µ = 0.23 mm^−^^1^
*c* = 13.6896 (12) Å	*T* = 100 K
β = 97.043 (3)°	Plate, colourless
*V* = 1788.9 (3) Å^3^	0.45 × 0.34 × 0.10 mm
*Z* = 4	

### Data collection

**Table:**

Bruker SMART APEXII DUO CCD area-detector diffractometer	8437 independent reflections
Radiation source: fine-focus sealed tube	6651 reflections with *I* > 2σ(*I*)
graphite	*R*_int_ = 0.038
φ and ω scans	θ_max_ = 36.2°, θ_min_ = 2.2°
Absorption correction: multi-scan (*SADABS*; Bruker, 2009)	*h* = −17→17
*T*_min_ = 0.904, *T*_max_ = 0.977	*k* = −14→20
21234 measured reflections	*l* = −22→22

### Refinement

**Table:**

Refinement on *F*^2^	Primary atom site location: structure-invariant direct methods
Least-squares matrix: full	Secondary atom site location: difference Fourier map
*R*[*F*^2^ > 2σ(*F*^2^)] = 0.050	Hydrogen site location: inferred from neighbouring sites
*wR*(*F*^2^) = 0.175	H-atom parameters constrained
*S* = 1.09	*w* = 1/[σ^2^(*F*_o_^2^) + (0.0914*P*)^2^ + 0.4364*P*] where *P* = (*F*_o_^2^ + 2*F*_c_^2^)/3
8437 reflections	(Δ/σ)_max_ < 0.001
248 parameters	Δρ_max_ = 0.75 e Å^−^^3^
0 restraints	Δρ_min_ = −0.39 e Å^−^^3^

### Special details

**Table:**

Experimental. The crystal was placed in the cold stream of an Oxford Cyrosystems Cobra open-flow nitrogen cryostat (Cosier & Glazer, 1986) operating at 100.0 (1) K.
Geometry. All e.s.d.'s (except the e.s.d. in the dihedral angle between two l.s. planes) are estimated using the full covariance matrix. The cell e.s.d.'s are taken into account individually in the estimation of e.s.d.'s in distances, angles and torsion angles; correlations between e.s.d.'s in cell parameters are only used when they are defined by crystal symmetry. An approximate (isotropic) treatment of cell e.s.d.'s is used for estimating e.s.d.'s involving l.s. planes.
Refinement. Refinement of *F*^2^ against ALL reflections. The weighted *R*-factor *wR* and goodness of fit *S* are based on *F*^2^, conventional *R*-factors *R* are based on *F*, with *F* set to zero for negative *F*^2^. The threshold expression of *F*^2^ > σ(*F*^2^) is used only for calculating *R*-factors(gt) *etc*. and is not relevant to the choice of reflections for refinement. *R*-factors based on *F*^2^ are statistically about twice as large as those based on *F*, and *R*- factors based on ALL data will be even larger.

### Fractional atomic coordinates and isotropic or equivalent isotropic displacement parameters (Å^2^)

**Table:**

	*x*	*y*	*z*	*U*_iso_*/*U*_eq_	Occ. (<1)
Cl1	1.44482 (3)	−0.12780 (3)	0.05943 (3)	0.02804 (10)	
O1	0.66151 (9)	0.53169 (7)	0.09366 (8)	0.02287 (19)	
O2	0.49447 (10)	0.47833 (8)	0.17101 (8)	0.0244 (2)	
N1	0.97430 (9)	0.22053 (7)	0.11935 (8)	0.01566 (17)	
N2	0.88205 (10)	0.08363 (8)	0.19214 (8)	0.01724 (18)	
C1	1.08637 (12)	−0.04972 (9)	0.10289 (9)	0.0184 (2)	
H1A	1.0056	−0.0819	0.0969	0.022*	
C2	1.19323 (13)	−0.10669 (10)	0.08102 (9)	0.0198 (2)	
H2A	1.1846	−0.1769	0.0607	0.024*	
C3	1.31246 (12)	−0.05767 (10)	0.08982 (9)	0.0193 (2)	
C4	1.32851 (12)	0.04663 (10)	0.12105 (10)	0.0204 (2)	
H4A	1.4096	0.0782	0.1271	0.025*	
C5	1.22138 (12)	0.10309 (9)	0.14312 (9)	0.0186 (2)	
H5A	1.2310	0.1730	0.1643	0.022*	
C6	1.09949 (11)	0.05585 (9)	0.13384 (8)	0.01582 (18)	
C7	0.98702 (11)	0.12116 (9)	0.14997 (8)	0.01543 (18)	
C8	0.85402 (10)	0.25092 (8)	0.14249 (8)	0.01417 (17)	
C9	0.79079 (11)	0.34784 (9)	0.12560 (8)	0.01504 (18)	
H9A	0.8286	0.4032	0.0947	0.018*	
C10	0.66963 (11)	0.35881 (8)	0.15650 (8)	0.01520 (18)	
C11	0.61175 (11)	0.27463 (9)	0.20282 (9)	0.0176 (2)	
H11A	0.5306	0.2846	0.2229	0.021*	
C12	0.67240 (12)	0.17741 (9)	0.21918 (9)	0.0187 (2)	
H12A	0.6338	0.1219	0.2493	0.022*	
C13	0.79468 (11)	0.16691 (9)	0.18806 (8)	0.01594 (19)	
C14	0.59808 (11)	0.46053 (9)	0.14271 (9)	0.01683 (19)	
C15	0.60047 (14)	0.63413 (10)	0.07588 (11)	0.0256 (3)	
H15A	0.6041	0.6740	0.1367	0.031*	
H15B	0.5107	0.6256	0.0489	0.031*	
C16	0.67404 (16)	0.69051 (12)	0.00368 (13)	0.0320 (3)	
H16A	0.6341	0.7577	−0.0131	0.048*	
H16B	0.6735	0.6483	−0.0547	0.048*	
H16C	0.7615	0.7016	0.0327	0.048*	
C17A	0.87973 (15)	−0.01159 (11)	0.25461 (12)	0.0170 (3)	0.855 (4)
H17A	0.9624	−0.0480	0.2532	0.020*	0.855 (4)
C18A	0.87305 (16)	0.02241 (14)	0.36090 (11)	0.0228 (3)	0.855 (4)
H18A	0.7882	0.0516	0.3663	0.027*	0.855 (4)
H18B	0.8847	−0.0393	0.4034	0.027*	0.855 (4)
C19A	0.97501 (17)	0.10445 (16)	0.39550 (12)	0.0268 (4)	0.855 (4)
H19A	0.9705	0.1207	0.4635	0.040*	0.855 (4)
H19B	0.9599	0.1676	0.3568	0.040*	0.855 (4)
H19C	1.0590	0.0768	0.3881	0.040*	0.855 (4)
C20A	0.77336 (17)	−0.08965 (12)	0.21576 (16)	0.0277 (4)	0.855 (4)
H20A	0.7817	−0.1070	0.1486	0.042*	0.855 (4)
H20B	0.6904	−0.0579	0.2194	0.042*	0.855 (4)
H20C	0.7812	−0.1529	0.2549	0.042*	0.855 (4)
C17B	0.9122 (10)	0.0082 (8)	0.2857 (7)	0.0179 (16)*	0.145 (4)
H17B	0.9924	−0.0301	0.2791	0.021*	0.145 (4)
C18B	0.8027 (10)	−0.0706 (8)	0.2733 (8)	0.027 (2)*	0.145 (4)
H18C	0.7234	−0.0335	0.2817	0.032*	0.145 (4)
H18D	0.8175	−0.1225	0.3257	0.032*	0.145 (4)
C19B	0.7823 (14)	−0.1313 (11)	0.1720 (11)	0.039 (3)*	0.145 (4)
H19D	0.7234	−0.1891	0.1764	0.059*	0.145 (4)
H19E	0.8636	−0.1582	0.1569	0.059*	0.145 (4)
H19F	0.7472	−0.0836	0.1210	0.059*	0.145 (4)
C20B	0.9247 (12)	0.0562 (10)	0.3823 (8)	0.027 (2)*	0.145 (4)
H20D	0.9400	0.0021	0.4316	0.040*	0.145 (4)
H20E	0.8466	0.0934	0.3909	0.040*	0.145 (4)
H20F	0.9959	0.1050	0.3886	0.040*	0.145 (4)

### Atomic displacement parameters (Å^2^)

**Table:**

	*U*^11^	*U*^22^	*U*^33^	*U*^12^	*U*^13^	*U*^23^
Cl1	0.02775 (17)	0.02678 (16)	0.03220 (17)	0.01538 (12)	0.01408 (13)	0.00528 (12)
O1	0.0225 (4)	0.0148 (4)	0.0334 (5)	0.0082 (3)	0.0119 (4)	0.0077 (3)
O2	0.0204 (4)	0.0219 (4)	0.0331 (5)	0.0080 (3)	0.0115 (4)	0.0027 (4)
N1	0.0143 (4)	0.0123 (3)	0.0216 (4)	0.0030 (3)	0.0068 (3)	0.0023 (3)
N2	0.0176 (4)	0.0122 (4)	0.0236 (4)	0.0040 (3)	0.0091 (3)	0.0050 (3)
C1	0.0196 (5)	0.0148 (4)	0.0209 (5)	0.0035 (4)	0.0035 (4)	0.0002 (4)
C2	0.0244 (5)	0.0156 (4)	0.0200 (5)	0.0062 (4)	0.0052 (4)	−0.0001 (4)
C3	0.0211 (5)	0.0189 (5)	0.0189 (5)	0.0093 (4)	0.0070 (4)	0.0035 (4)
C4	0.0175 (5)	0.0192 (5)	0.0256 (5)	0.0044 (4)	0.0068 (4)	0.0022 (4)
C5	0.0178 (5)	0.0153 (4)	0.0237 (5)	0.0031 (4)	0.0071 (4)	0.0010 (4)
C6	0.0171 (4)	0.0135 (4)	0.0177 (4)	0.0043 (3)	0.0054 (4)	0.0023 (3)
C7	0.0156 (4)	0.0130 (4)	0.0186 (4)	0.0034 (3)	0.0059 (3)	0.0023 (3)
C8	0.0140 (4)	0.0114 (4)	0.0179 (4)	0.0019 (3)	0.0054 (3)	0.0021 (3)
C9	0.0155 (4)	0.0121 (4)	0.0181 (4)	0.0025 (3)	0.0046 (3)	0.0019 (3)
C10	0.0155 (4)	0.0131 (4)	0.0175 (4)	0.0033 (3)	0.0042 (3)	0.0005 (3)
C11	0.0158 (4)	0.0169 (4)	0.0212 (5)	0.0025 (4)	0.0064 (4)	0.0017 (4)
C12	0.0169 (5)	0.0161 (4)	0.0246 (5)	0.0019 (4)	0.0089 (4)	0.0045 (4)
C13	0.0162 (4)	0.0136 (4)	0.0191 (4)	0.0033 (3)	0.0064 (4)	0.0032 (3)
C14	0.0170 (5)	0.0149 (4)	0.0189 (4)	0.0040 (3)	0.0034 (4)	0.0007 (3)
C15	0.0283 (6)	0.0170 (5)	0.0334 (6)	0.0121 (4)	0.0118 (5)	0.0070 (4)
C16	0.0335 (7)	0.0215 (6)	0.0440 (8)	0.0110 (5)	0.0171 (6)	0.0111 (6)
C17A	0.0178 (6)	0.0125 (5)	0.0212 (6)	0.0013 (4)	0.0039 (5)	0.0058 (5)
C18A	0.0219 (7)	0.0279 (7)	0.0197 (6)	0.0051 (6)	0.0064 (5)	0.0090 (5)
C19A	0.0267 (8)	0.0356 (9)	0.0180 (6)	0.0097 (7)	0.0016 (5)	−0.0007 (6)
C20A	0.0229 (7)	0.0157 (6)	0.0456 (11)	−0.0037 (5)	0.0083 (7)	0.0041 (6)

### Geometric parameters (Å, °)

**Table:**

Cl1—C3	1.7332 (12)	C15—C16	1.503 (2)
O1—C14	1.3433 (15)	C15—H15A	0.9700
O1—C15	1.4486 (15)	C15—H15B	0.9700
O2—C14	1.2131 (15)	C16—H16A	0.9600
N1—C7	1.3240 (14)	C16—H16B	0.9600
N1—C8	1.3854 (14)	C16—H16C	0.9600
N2—C7	1.3828 (15)	C17A—C18A	1.527 (2)
N2—C13	1.3879 (14)	C17A—C20A	1.530 (2)
N2—C17A	1.4769 (16)	C17A—H17A	0.9800
N2—C17B	1.595 (9)	C18A—C19A	1.518 (3)
C1—C2	1.3897 (17)	C18A—H18A	0.9700
C1—C6	1.3998 (16)	C18A—H18B	0.9700
C1—H1A	0.9300	C19A—H19A	0.9600
C2—C3	1.3812 (19)	C19A—H19B	0.9600
C2—H2A	0.9300	C19A—H19C	0.9600
C3—C4	1.3879 (18)	C20A—H20A	0.9600
C4—C5	1.3895 (17)	C20A—H20B	0.9600
C4—H4A	0.9300	C20A—H20C	0.9600
C5—C6	1.3960 (17)	C17B—C20B	1.447 (15)
C5—H5A	0.9300	C17B—C18B	1.508 (15)
C6—C7	1.4725 (15)	C17B—H17B	0.9800
C8—C9	1.3956 (15)	C18B—C19B	1.576 (18)
C8—C13	1.4112 (15)	C18B—H18C	0.9700
C9—C10	1.3881 (16)	C18B—H18D	0.9700
C9—H9A	0.9300	C19B—H19D	0.9600
C10—C11	1.4104 (16)	C19B—H19E	0.9600
C10—C14	1.4854 (15)	C19B—H19F	0.9600
C11—C12	1.3863 (16)	C20B—H20D	0.9600
C11—H11A	0.9300	C20B—H20E	0.9600
C12—C13	1.3998 (16)	C20B—H20F	0.9600
C12—H12A	0.9300		
			
C14—O1—C15	116.59 (10)	O1—C15—C16	106.49 (11)
C7—N1—C8	104.38 (9)	O1—C15—H15A	110.4
C7—N2—C13	105.87 (9)	C16—C15—H15A	110.4
C7—N2—C17A	126.20 (10)	O1—C15—H15B	110.4
C13—N2—C17A	125.61 (10)	C16—C15—H15B	110.4
C7—N2—C17B	116.8 (4)	H15A—C15—H15B	108.6
C13—N2—C17B	123.2 (3)	C15—C16—H16A	109.5
C2—C1—C6	120.43 (12)	C15—C16—H16B	109.5
C2—C1—H1A	119.8	H16A—C16—H16B	109.5
C6—C1—H1A	119.8	C15—C16—H16C	109.5
C3—C2—C1	119.14 (11)	H16A—C16—H16C	109.5
C3—C2—H2A	120.4	H16B—C16—H16C	109.5
C1—C2—H2A	120.4	N2—C17A—C18A	109.21 (12)
C2—C3—C4	121.67 (11)	N2—C17A—C20A	112.67 (14)
C2—C3—Cl1	119.06 (10)	C18A—C17A—C20A	113.35 (13)
C4—C3—Cl1	119.26 (10)	N2—C17A—H17A	107.1
C3—C4—C5	118.92 (12)	C18A—C17A—H17A	107.1
C3—C4—H4A	120.5	C20A—C17A—H17A	107.1
C5—C4—H4A	120.5	C19A—C18A—C17A	112.15 (12)
C4—C5—C6	120.60 (11)	C19A—C18A—H18A	109.2
C4—C5—H5A	119.7	C17A—C18A—H18A	109.2
C6—C5—H5A	119.7	C19A—C18A—H18B	109.2
C5—C6—C1	119.22 (10)	C17A—C18A—H18B	109.2
C5—C6—C7	118.75 (10)	H18A—C18A—H18B	107.9
C1—C6—C7	121.88 (11)	C20B—C17B—C18B	111.2 (9)
N1—C7—N2	113.76 (9)	C20B—C17B—N2	118.1 (8)
N1—C7—C6	122.25 (10)	C18B—C17B—N2	103.4 (8)
N2—C7—C6	123.86 (10)	C20B—C17B—H17B	107.9
N1—C8—C9	128.88 (10)	C18B—C17B—H17B	107.9
N1—C8—C13	110.60 (9)	N2—C17B—H17B	107.9
C9—C8—C13	120.52 (10)	C17B—C18B—C19B	116.0 (9)
C10—C9—C8	117.80 (10)	C17B—C18B—H18C	108.3
C10—C9—H9A	121.1	C19B—C18B—H18C	108.3
C8—C9—H9A	121.1	C17B—C18B—H18D	108.3
C9—C10—C11	121.19 (10)	C19B—C18B—H18D	108.3
C9—C10—C14	120.64 (10)	H18C—C18B—H18D	107.4
C11—C10—C14	118.17 (10)	C18B—C19B—H19D	109.5
C12—C11—C10	121.83 (10)	C18B—C19B—H19E	109.5
C12—C11—H11A	119.1	H19D—C19B—H19E	109.5
C10—C11—H11A	119.1	C18B—C19B—H19F	109.5
C11—C12—C13	116.72 (10)	H19D—C19B—H19F	109.5
C11—C12—H12A	121.6	H19E—C19B—H19F	109.5
C13—C12—H12A	121.6	C17B—C20B—H20D	109.5
N2—C13—C12	132.68 (10)	C17B—C20B—H20E	109.5
N2—C13—C8	105.39 (9)	H20D—C20B—H20E	109.5
C12—C13—C8	121.93 (10)	C17B—C20B—H20F	109.5
O2—C14—O1	123.27 (11)	H20D—C20B—H20F	109.5
O2—C14—C10	124.96 (11)	H20E—C20B—H20F	109.5
O1—C14—C10	111.77 (10)		
			
C6—C1—C2—C3	−0.27 (18)	C17B—N2—C13—C12	−41.3 (5)
C1—C2—C3—C4	0.76 (19)	C7—N2—C13—C8	0.12 (13)
C1—C2—C3—Cl1	−178.33 (9)	C17A—N2—C13—C8	163.64 (13)
C2—C3—C4—C5	−0.55 (19)	C17B—N2—C13—C8	138.5 (5)
Cl1—C3—C4—C5	178.54 (10)	C11—C12—C13—N2	179.91 (13)
C3—C4—C5—C6	−0.15 (19)	C11—C12—C13—C8	0.17 (18)
C4—C5—C6—C1	0.63 (18)	N1—C8—C13—N2	−0.11 (13)
C4—C5—C6—C7	−174.93 (11)	C9—C8—C13—N2	179.26 (10)
C2—C1—C6—C5	−0.42 (17)	N1—C8—C13—C12	179.69 (11)
C2—C1—C6—C7	175.00 (11)	C9—C8—C13—C12	−0.94 (18)
C8—N1—C7—N2	0.02 (14)	C15—O1—C14—O2	0.54 (19)
C8—N1—C7—C6	176.15 (11)	C15—O1—C14—C10	179.96 (11)
C13—N2—C7—N1	−0.09 (14)	C9—C10—C14—O2	−176.84 (12)
C17A—N2—C7—N1	−163.48 (13)	C11—C10—C14—O2	2.36 (19)
C17B—N2—C7—N1	−141.6 (4)	C9—C10—C14—O1	3.75 (16)
C13—N2—C7—C6	−176.15 (11)	C11—C10—C14—O1	−177.05 (11)
C17A—N2—C7—C6	20.5 (2)	C14—O1—C15—C16	−168.74 (13)
C17B—N2—C7—C6	42.4 (5)	C7—N2—C17A—C18A	108.23 (14)
C5—C6—C7—N1	38.83 (17)	C13—N2—C17A—C18A	−52.01 (18)
C1—C6—C7—N1	−136.61 (12)	C17B—N2—C17A—C18A	38.7 (9)
C5—C6—C7—N2	−145.43 (12)	C7—N2—C17A—C20A	−124.87 (14)
C1—C6—C7—N2	39.13 (17)	C13—N2—C17A—C20A	74.90 (18)
C7—N1—C8—C9	−179.25 (12)	C17B—N2—C17A—C20A	165.6 (10)
C7—N1—C8—C13	0.06 (13)	N2—C17A—C18A—C19A	−50.27 (16)
N1—C8—C9—C10	−179.71 (11)	C20A—C17A—C18A—C19A	−176.79 (13)
C13—C8—C9—C10	1.04 (17)	C7—N2—C17B—C20B	92.8 (8)
C8—C9—C10—C11	−0.44 (17)	C13—N2—C17B—C20B	−41.5 (10)
C8—C9—C10—C14	178.73 (10)	C17A—N2—C17B—C20B	−145.1 (15)
C9—C10—C11—C12	−0.32 (18)	C7—N2—C17B—C18B	−144.0 (5)
C14—C10—C11—C12	−179.51 (11)	C13—N2—C17B—C18B	81.8 (7)
C10—C11—C12—C13	0.44 (18)	C17A—N2—C17B—C18B	−21.9 (7)
C7—N2—C13—C12	−179.65 (13)	C20B—C17B—C18B—C19B	−178.0 (9)
C17A—N2—C13—C12	−16.1 (2)	N2—C17B—C18B—C19B	54.3 (10)

### Hydrogen-bond geometry (Å, °)

**Table:**

Cg1 is the centroid of the N1/C7/N2/C13/C8 ring.

**Table:**

*D*—H···*A*	*D*—H	H···*A*	*D*···*A*	*D*—H···*A*
C12—H12A···O2^i^	0.93	2.57	3.4994 (16)	174
C16—H16A···Cl1^ii^	0.96	2.73	3.4651 (17)	134
C19A—H19B···Cg1	0.96	2.71	3.3457 (18)	124

Symmetry codes: (i) −*x*+1, *y*−1/2, −*z*+1/2; (ii) *x*−1, *y*+1, *z*.
